# Lagebild Bevölkerungsverhalten für ein effektives Krisenmanagement

**DOI:** 10.1007/s00103-022-03583-2

**Published:** 2022-09-16

**Authors:** Nathalie Schopp, Charline Schüler, Volker Tondorf, Lynn Schüller

**Affiliations:** Referat I.3 – Psychosoziales Krisenmanagement, Bundesamt für Bevölkerungsschutz und Katastrophenhilfe, Provinzialstr. 93, 53127 Bonn, Deutschland

**Keywords:** Bevölkerung, Krisenstab, Resilienz, Verhaltensforschung, Katastrophenschutz, Population, Crisis response, Resilience, Behavioural science, Civil protection

## Abstract

Wie elementar sozialwissenschaftliche Erkenntnisse für die Krisenbewältigung sind, hat besonders die Coronavirus-Pandemie gezeigt, in der das Bevölkerungsverhalten ein zentraler Dreh- und Angelpunkt für den gesamten Krisenverlauf war und ist. Derzeit wird das Wissen zum Bevölkerungsverhalten in Lagebildern häufig nicht berücksichtigt und kann daher durch Krisenstäbe nicht in ausreichendem Maße genutzt werden.

Dies soll das Projekt „Lagebild Bevölkerungsverhalten für ein effektives staatliches Krisenmanagement (LB BevV)“ ändern, welches im Referat Psychosoziales Krisenmanagement im Bundesamt für Bevölkerungsschutz und Katastrophenhilfe (BBK) angesiedelt ist. Dieses Projekt bildet den Fokus dieses Artikels. Ein strategisches Ziel des Projekts ist es, Wissen zum Bevölkerungsverhalten stärker in das staatliche Krisenmanagement zu integrieren. Damit einhergehend gilt es, die differenzierte Einbeziehung der Bevölkerung für die Bewältigung von Krisen und Katastrophen zu standardisieren. Darüber hinaus kann mithilfe des Lagebilds Bevölkerungsverhalten staatliches Handeln die Bewältigungskompetenzen von Betroffenen besser unterstützen, weil so z. B. die Bedarfe, Bedürfnisse und Ressourcen der Bevölkerung aufgezeigt werden können. Dadurch können staatliche Maßnahmen gezielt angepasst werden.

Innerhalb des Projekts LB BevV sind weitere BBK-geförderte Forschungsprojekte angesiedelt mit dem Ziel, den menschzentrierten Bevölkerungsschutz mithilfe von wissenschaftlichen Erkenntnissen zu stärken. Die Ergebnisse der Forschungsprojekte fließen in das Projekt LB BevV mit ein. Das Lagebild wurde in einer Testphase alle 2 Wochen auf Basis von Daten und Erkenntnissen aus öffentlich zugänglichen Studien erstellt und verschiedenen Bedarfsträgern zur Verfügung gestellt.

## Abstract (en)

The Coronavirus pandemic, in which public response was and still is central to damage control, has demonstrated how significant psychological and social science insights are for crisis management. Currently, knowledge of public response is often not incorporated into situational reports and, therefore, cannot be sufficiently considered by crisis management teams.

With the intent of supporting change, the project “Situational Awareness of Public Response”, conducted by the Division of Psychosocial Crisis Management within the Federal Office of Civil Protection and Disaster Assistance, is currently designing a framework for including public response knowledge in crisis management decision-making. The main focus of this paper is this project. A strategic objective is to integrate public response knowledge more strongly into governmental crisis management. On the basis of evidence-based assessments, the needs and demands of the population can be identified and coping capacities can be better supported by crisis management measures.

Several BBK-funded research projects are affiliated to the aforementioned main project, with the objective of strengthening human-centric civil protection. In a test phase, the situational picture of public response was compiled every two weeks on the basis of data and findings from publicly available studies and made available to various stakeholders.

## Einleitung

Die Coronavirus-Pandemie hat sehr deutlich vor Augen geführt, dass das Bevölkerungsverhalten in Krisen- und Katastrophenlagen ein zentraler Dreh- und Angelpunkt für den gesamten Krisenverlauf ist – von der Schadensbewältigung bis hin zur Prävention weiterer Schäden. Ob es um die Einhaltung von Hygienemaßnahmen, Abstandsregelungen oder weiteren Maßnahmen geht – die wissenschaftlich fundierten Kenntnisse zu sozialen Dynamiken, Risikowahrnehmung, Informationsbedarfen und Selbstschutzkompetenzen bilden eine wichtige Entscheidungsgrundlage für effektives und effizientes Krisenmanagement. Auch die Ereignisse um die Hochwasserlage im Sommer 2021 in Westdeutschland legen nahe: Die Effektivität des Krisenmanagements steigt, wenn Bewältigungsstrategien und Informationsbedarfe der Bevölkerung bekannt sind und berücksichtigt werden.

Derzeit wird das Wissen zum Bevölkerungsverhalten in Lagebildern häufig nicht berücksichtigt und kann daher durch Krisenstäbe nicht in ausreichendem Maße genutzt werden. Das Referat „Psychosoziales Krisenmanagement“ im Bundesamt für Bevölkerungsschutz und Katastrophenhilfe (BBK) hat das Projekt „Lagebild Bevölkerungsverhalten für ein effektives staatliches Krisenmanagement (LB BevV)“ ins Leben gerufen, um diese Lücke zu schließen.

Der vorliegende Beitrag beschäftigt sich hauptsächlich mit der näheren Betrachtung dieses Projektes. Neben der Vorstellung des Projektvorhabens wird zu Beginn der Begriff „Bevölkerungsverhalten“ definiert. Anschließend folgt die Vorstellung weiterer BBK-geförderter Forschungsprojekte, deren Ergebnisse in das Projekt LB BevV mit einfließen. Abschließend wird das „Lagebild Bevölkerungsverhalten“ betrachtet, dass die Projektgruppe in einer Testphase im zweiwöchigen Turnus erstellt und verschiedenen Bedarfsträgern zugeliefert hat.

## Projekt „Lagebild Bevölkerungsverhalten“

Schon seit vielen Jahren arbeitet das Referat „Psychosoziales Krisenmanagement“ im BBK zu Bedarfen und Bedürfnissen der Bevölkerung in Krisen und Katastrophen. Seit 2021 führt es das Projekt „Lagebild Bevölkerungsverhalten für ein effektives staatliches Krisenmanagement“ durch mit dem Ziel, Wissen zum Bevölkerungsverhalten besser in das staatliche Krisenmanagement zu integrieren und dieses im Krisen- und Katastrophenfall anwendbar zu machen [1]. Unabdingbar für die Lageübersicht und eine nachfolgende Entscheidungsfindung in Krisenstäben sind regelmäßige Lageberichte, die sich zu einem Lagebild formen. Innerhalb des Projekts LB BevV wurde ein solches „Lagebild Bevölkerungsverhalten“ entwickelt, um Krisenstäben eine fundierte Ergänzung der vorliegenden Lageinformationen bieten zu können. Darauf aufbauend können die Bewältigungskompetenzen von Betroffenen besser unterstützt und ggf. die Akzeptanz für staatliche Empfehlungen erhöht werden.

Im Rahmen des Projekts ist das Bevölkerungsverhalten ein zentraler Gegenstand und wird wie folgt definiert:

*Bevölkerungsverhalten* ist ein übergeordneter Begriff für das Verhalten und Erleben von Menschen, die einer bestimmten Bevölkerungsgruppe zugerechnet werden. Unter Bevölkerungsverhalten wird hier (*anders als in der Psychologie üblich*) nicht nur das beobachtbare Verhalten verstanden, sondern auch kognitive, emotionale, motivationale sowie soziale Prozesse.

Im allgemeinen Sprachgebrauch wird häufig von „*der* Bevölkerung“ gesprochen. Es ist jedoch zu beachten, dass sich eine Gesellschaft aus unterschiedlichen Individuen, Gruppen und Teilgesellschaften zusammensetzt und damit auch das Verhalten der Bevölkerung in der Regel eine erhebliche Varianz aufweist. Das Bevölkerungsverhalten auf individueller, sozialer und gesellschaftlicher Ebene wird von verschiedenen Merkmalen und Faktoren beeinflusst. Soziodemografische Merkmale (Alter, Geschlecht etc.) spielen ebenso eine Rolle wie soziale Lebensräume und die Sozialstruktur eines Gebietes (Bildung, Migrationsgeschichte, Einkommen).

Im Bevölkerungsschutz wird das Bevölkerungsverhalten vor allem im Zusammenhang mit der Vorbereitung auf und der Bewältigung von Krisen und Katastrophen betrachtet. Ziel ist es nicht nur, das Verhalten der Bevölkerung zu beschreiben und zu erklären, sondern dieses ggf. auch prognostizieren zu können sowie vorhandene gesellschaftliche Ressourcen zu erfassen und Unterstützungsangebote vorzubereiten. Besonderes Augenmerk gilt der Identifizierung all jener Faktoren, die einen unterstützenden Einfluss auf die Bewältigungskompetenz im Hinblick auf Krisen haben.^1^

Während der Coronavirus-Pandemie ist deutlich geworden, wie wichtig die Berücksichtigung der gesellschaftlichen Perspektive als integraler Bestandteil im Krisenmanagement ist. Während des Hochwassers 2021 in Westdeutschland sind erneut das große Potenzial und Engagement der Spontanhelfenden in den Fokus gerückt. Dennoch wird das Selbsthilfepotenzial der Bevölkerung bislang zu wenig genutzt und in die Krisenbewältigung miteinbezogen. Faktoren dafür sind z. B. Alltagsmythen in Bezug auf das Verhalten der Bevölkerung in Krisen- und Katastrophenzeiten, die sich hartnäckig halten. Obwohl seit Beginn der katastrophensoziologischen Forschung immer wieder nachgewiesen wurde, dass während und nach großen Krisen und Katastrophen soziales, unterstützendes Verhalten vorherrscht [2–4], ist die Verankerung stereotyper Vorstellungen zu Massenpaniken und sozialen Zusammenbrüchen als typische Reaktion auf akute Krisen nach wie vor stark ausgeprägt [4, 5]. Damit einhergehend ist ein strategisches Ziel des Projektes LB BevV, die differenzierte Einbeziehung der Bevölkerung in all ihrer Heterogenität bei der Bewältigung von Krisen und Katastrophen zu standardisieren. Die Notwendigkeit eines solchen Strategie- bzw. Paradigmenwechsels im Bevölkerungsschutz wird auch von neuen Forschungen hervorgehoben [4, 6–8].

## Begleitforschung

Innerhalb des Projekts LB BevV sind weitere BBK-geförderte Forschungsprojekte angesiedelt, mit dem Ziel, einen menschzentrierten Bevölkerungsschutz wissenschaftlich zu stärken. Die Ergebnisse der folgenden Begleitforschungsprojekte fließen in das Projekt LB BevV mit ein.

Einen wichtigen Beitrag leistet die Universität Jena mit dem im Februar 2020 begonnenen Ressortforschungsprojekt „Das Lagebild Bevölkerungsverhalten in der Stabsarbeit (LaBS)“. Das vom Bundesministerium des Innern und für Heimat (BMI) und dem BBK geförderte Vorhaben hat zum Ziel, erstmalig die psychosoziale Dimension systematisch in die Stabsarbeit zu integrieren. Dafür wird zunächst definiert, was ein Lagebild Bevölkerungsverhalten beinhalten sollte und welche Informationsquellen zur Erstellung genutzt werden können. Es wird außerdem untersucht, ob und wie das Lagebild Bevölkerungsverhalten bei der Konzeption und Durchführung von Stabsübungen einbezogen werden kann. Die Ergebnisse der Analyse werden für (Verwaltungs‑)Stäbe nutzbar gemacht und sollen die eigenständige Erstellung eines Lagebilds ermöglichen.

Ein weiteres Forschungsprojekt, das im August 2021 startete, wird von der Universität Wuppertal durchgeführt und widmet sich den kurz- und mittelfristigen sozialen Anpassungsprozessen der Bevölkerung in unterschiedlichen Zivil- und Katastrophenlagen. Ziel ist die „Entwicklung eines Sozialkapital-Radars für den sozialraumorientierten Bevölkerungsschutz (Sokapi-R)“, mit dem sich die soziale Unterstützungsbereitschaft in verschiedenen Krisen und Katastrophenlagen kleinräumig identifizieren und nachvollziehen lässt. Hoher Zusammenhalt und starkes Vertrauen bilden als Sozialkapital das Fundament einer resilienten Gemeinschaft. Am Beispiel der Stadt Wuppertal wird dabei zunächst der Zusammenhang von sozialen Strukturen und lokalem Sozialkapital operationalisiert und im Rahmen einer quantitativen, mehrsprachig umgesetzten Bevölkerungsbefragung empirisch validiert. Zusammen mit Sozialdaten der Stadt Wuppertal wird auf dieser Grundlage ein interaktives Lagebild zum Bevölkerungsverhalten entwickelt. Das Sozialkapital-Radar soll ein Tool werden, mit dessen Hilfe soziale Unterstützungsgemeinschaften im sozialen Nahraum identifiziert werden können sowie ein bedarfs- und ressourcenorientiertes Krisenmanagement umgesetzt werden kann [9].

Das Forschungsprojekt „Systematische Analyse der Kommunikation in sozialen Medien zur Anfertigung Psychosozialer Lagebilder in Krisen und Katastrophen (#SOSMAP)“ wird ebenfalls von der Universität Wuppertal durchgeführt und startet im Herbst 2022. Das Projekt wird Rahmenempfehlungen für die Auswertung sozialer Medien im Hinblick auf psychosoziale Bedarfe der Bevölkerung entwickeln. Darüber hinaus soll ein an die Anforderungen von Entscheidungsträgern angepasstes Kategorisierungsraster zur Aus- und Bewertung psychosozialer Bedarfe und Ressourcen der Bevölkerung entwickelt werden.

Neben diesen Forschungsprojekten gibt es auch erste erfolgreiche praktische Ansätze für ein Lagebild Bevölkerungsverhalten im kommunalen Bereich: In der Coronavirus-Lage integrierte z. B. der Corona-Krisenstab der Stadt Mülheim an der Ruhr (Nordrhein-Westfalen) ein Sachgebiet, das später „Stabsstelle kommunales psychosoziales Krisenmanagement“ genannt wurde. Von der Stabsstelle, die bis September 2021 dem Amt für Brandschutz, Rettungsdienst, Zivil- und Katastrophenschutz angegliedert war, wurden Strukturen, Maßnahmen und Angebote entwickelt, um die einzelnen Bevölkerungsgruppen bei der Bewältigung des Pandemiegeschehens zu begleiten und zu unterstützen. Dieses Engagement soll im Rahmen einer „Dokumentation des kommunalen psychosozialen Krisenmanagements im Rahmen der Coronavirus-Pandemie (DokoPsy)“ systematisch aufbereitet und dokumentiert werden – damit die gesammelten Erfahrungen ggf. auch für andere Krisenstäbe bzw. andere Gefahren- und Schadenslagen nutzbar sind [10].

## Produkt: Lagebild Bevölkerungsverhalten

Von Dezember 2021 bis August 2022 wurde im Projekt LB BevV ein eigenes Lagebild zur Coronavirus-Pandemie sowie zum russischen Angriffskrieg auf die Ukraine konzipiert. Dieses neue sogenannte Lagebild Bevölkerungsverhalten wurde unterschiedlichen Bedarfsträgern testweise zur Verfügung gestellt: dem Gemeinsamen Melde- und Lagezentrum (GMLZ) BBK-intern, dem neuen Gemeinsamen Kompetenzzentrum Bevölkerungsschutz beim BBK (GeKoB) für das Gemeinsame Lagebild Bevölkerungsschutz von Bund und Ländern sowie zeitweise auch dem Krisenstab des Bundeskanzleramtes.

**Figure 1:**
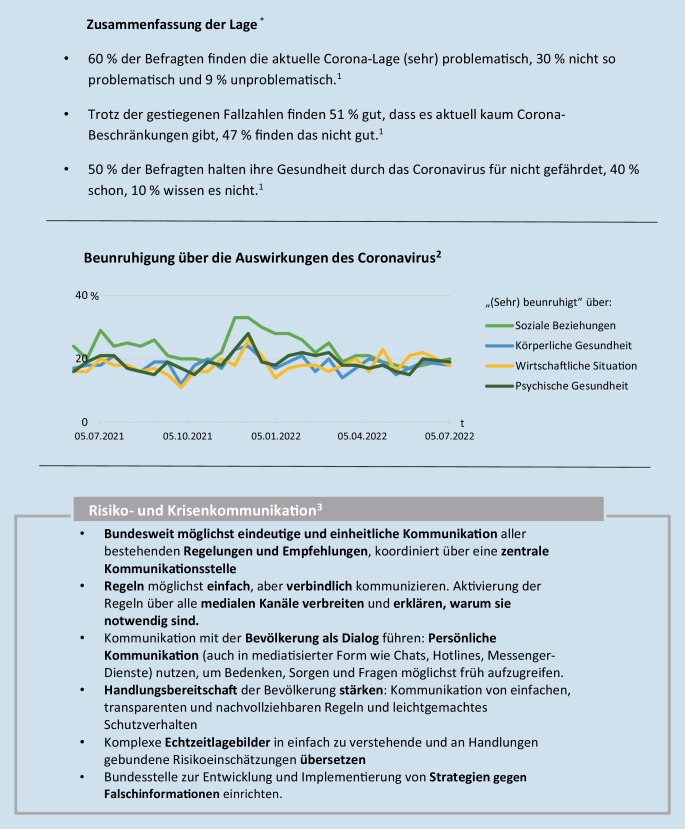

In Abb. 1 wird exemplarisch ein Teil des Lagebilds Bevölkerungsverhalten zur Coronavirus-Pandemie dargestellt, der am 22.07.2022 erstellt wurde. Im ersten Abschnitt werden die wichtigsten Informationen zusammengefasst. Anschließend wird die Beunruhigung über die Auswirkung des Coronavirus dargestellt. Abschließend werden verschiedene Handlungsempfehlungen für eine effektive Risiko- und Gesundheitskommunikation aufgezeigt. Die jeweiligen Daten – im Hinblick auf die Pandemie sowie im Hinblick auf den russischen Angriffskrieg auf die Ukraine – stammten dabei aus unterschiedlichen Quellen. Während der Coronavirus-Pandemie wurden in unterschiedlichen quantitativen Studien über Bevölkerungsbefragungen erstmals wissenschaftlich systematisch Daten zu Risikowahrnehmung, Akzeptanz, Bewältigungsverhalten und der Bewertung des staatlichen Krisenmanagements erhoben.

Eine dieser Studien ist das Gemeinschaftsprojekt „Ergebnisse aus dem *CO*VID-19 *S*napshot *MO*nitoring (*COSMO)*: Die psychologische Lage“, welches in Zusammenarbeit mit mehreren Projektpartnern durchgeführt wurde: der Universität Erfurt, dem Robert Koch-Institut, der Bundeszentrale für gesundheitliche Aufklärung, dem Leibniz-Institut für Psychologie, dem Science Media Center, dem Bernhard-Nocht-Institut für Tropenmedizin und Yale Institute for Global Health. In regelmäßigen Abständen wurden von März 2020 bis März 2022 bundesweite Erhebungen durchgeführt. Die Schwerpunkte lagen dabei auf Risikowahrnehmung, Lebenszufriedenheit, Belastung und psychische Ressourcen, Wissen und Verhalten, Akzeptanz und Ablehnung politischer Maßnahmen, Vertrauen und Demonstrationsbereitschaft, Einstellung und Akzeptanz zu Impfungen sowie Pandemiemüdigkeit [11].

Eine weitere Studie wird federführend vom Robert Koch-Institut durchgeführt, das „COVID-19 Impfquoten-Monitoring in Deutschland (COVIMO)“. Dabei werden regelmäßig Daten zu Impfverhalten, Impfbereitschaft und -akzeptanz in Deutschland erhoben, aufbereitet und veröffentlicht [12]. Auch der „BfR-Corona-Monitor“ des Bundesinstituts für Risikobewertung (BfR) berichtet regelmäßig über die Wahrnehmung der Pandemielage in der Bevölkerung. Unter anderem wird dabei zu wahrgenommener Informiertheit, der Einschätzung der Medienberichterstattung sowie dem Risikobewusstsein und den Schutzmaßnahmen bundesweit befragt [13].

Neben der Systematisierung eines Lagebilds Bevölkerungsverhalten und der Kategorisierung möglicher Inhalte stellen die Quellensuche und die Datengenerierung große Herausforderungen dar. Durch die pandemische Dauerlage standen die 3 oben genannten Studien zur Verfügung, die durch ihre regelmäßigen Erhebungen zahlreiche Trends, Tendenzen und Entwicklungen – beispielsweise zur Impfbereitschaft – darstellen konnten. Es ist jedoch eher unüblich, dass ereignisbezogene Daten z. B. zur Risikowahrnehmung erhoben werden, was besonders die Erstellung des Lagebilds zum russischen Angriffskrieg auf die Ukraine erschwert hat. Aus diesem Grund hat das Projekts LB BevV eine eigene repräsentative Studie mit regelmäßigen Erhebungen von Daten zum Bevölkerungsverhalten in Deutschland durchführen lassen.

Die o. g. Studien weisen Limitationen auf, die u. a. aus der Zusammensetzung der Stichproben resultieren. Es ist essenziell, auch solche Gruppen zu erfassen, die häufig in Erhebungen unterrepräsentiert sind, z. B. Menschen, die aufgrund von Sprachbarrieren nicht teilnehmen (können). Dies muss grundsätzlich bei allen Datenerhebungen zum Bevölkerungsverhalten berücksichtigt werden. Gleichzeitig lag die Auswertung der Daten häufig erst 1–2 Wochen nach Erhebung vor. Für das Krisenmanagement ist das je nach Lage eine zeitliche Verzögerung, die den Nutzen der Datenerhebung infrage stellen kann.

Auch soziale Medien fungieren als Datenquelle (siehe Forschungsprojekt „#SOSMAP“). Besonders hierbei muss bedacht werden, dass in sozialen Medien häufig einer begrenzten Auswahl an Themen eine überhöhte Aufmerksamkeit zuteilwird. Die gesellschaftlichen Gruppen oder Akteur:innen, die für ihre Meinungen in den sozialen Medien eine große Öffentlichkeit generieren können, müssen in ihrer Dimension richtig eingeordnet werden. Grundsätzlich werden methodische Defizite und die notwendige Selektion der Daten jede Darstellung von Bevölkerungsverhalten subjektiv prägen. Diese Limitationen bedürfen einer ständigen kritischen Auseinandersetzung.

## Fazit

Lagebilder schärfen das Lageverständnis und sind die elementare Grundlage der Entscheidungsfindung im Krisenfall. Auch jenseits von Krisen sind sie eine wichtige Basis, um einen aktuellen Überblick über das jeweils relevante Themenfeld zu gewinnen. Gegenwärtig fehlt die systematische Einbeziehung von Daten zum Bevölkerungsverhalten. Dadurch gibt es oft wenig Wissen über die Bevölkerung in Krisen. Auch Feedback zu bereits umgesetzten Maßnahmen, das man braucht, um das Krisenmanagement ggf. anzupassen, kann über solche Lagebilder dargestellt werden.

Das Projekt „Lagebild Bevölkerungsverhalten für ein effektives staatliches Krisenmanagement“, durchgeführt vom Bundesamt für Bevölkerungsschutz und Katastrophenhilfe, will diese Lücke weiter schließen und Wissen zum Bevölkerungsverhalten stärker in das staatliche Krisenmanagement integrieren. Damit einhergehend gilt es, die differenzierte Einbeziehung der Bevölkerung für die Bewältigung von Krisen und Katastrophen zu standardisieren. Mithilfe des Lagebilds Bevölkerungsverhalten werden Bedarfe, Bedürfnisse und Ressourcen der Bevölkerung aufgezeigt. So können die Bewältigungskompetenzen von Betroffenen besser unterstützt werden.

### Infobox Fachkongress „Mensch und Gesellschaft im Bevölkerungsschutz“ 2023

Mit Fragen u. a. zur Datengenerierung wird sich auch der Fachkongress „Mensch und Gesellschaft im Bevölkerungsschutz“ vom 25.09. bis zum 27.09.2023 beschäftigen, eine Veranstaltung der Bundesakademie für Bevölkerungsschutz und Zivile Verteidigung (BABZ). Die Themenschwerpunkte sind dabei Lagebild Bevölkerungsverhalten, Warnung und Krisenmanagement, Social Media im Bevölkerungsschutz und psychosoziale Notfallversorgung.

## Footnotes

1 BBK (2022 – unveröffentlicht). Definition Bevölkerungsverhalten.
