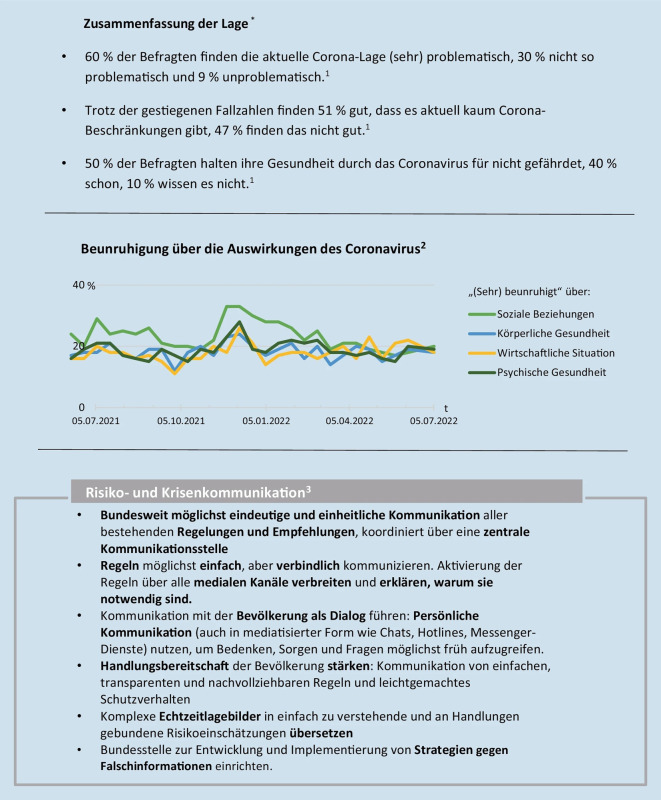# Erratum zu: Lagebild Bevölkerungsverhalten für ein effektives Krisenmanagement

**DOI:** 10.1007/s00103-022-03601-3

**Published:** 2022-11-22

**Authors:** Nathalie Schopp, Charline Schüler, Volker Tondorf, Lynn Schüller

**Affiliations:** Referat I.3 – Psychosoziales Krisenmanagement, Bundesamt für Bevölkerungsschutz und Katastrophenhilfe, Provinzialstr. 93, 53127 Bonn, Deutschland

**Erratum zu:**

**Bundesgesundheitsbl 2022**

10.1007/s00103-022-03583-2

In der ursprünglichen Originalversion des Artikels wurden die Legenden zu den inkludierten Fußnoten in der Abbildung (Abb. 1) nicht in der Abbildungsunterschrift umgesetzt. Die korrigierte Version der Abbildungsunterschrift zusammen mit Abb. 1 ist im Folgenden abgebildet.

**Figure 1:**